# Does the Number of Interbody Devices Affect the Fusion Outcome in Short-Segment Posterior Lumbar Fusion?

**DOI:** 10.7759/cureus.50113

**Published:** 2023-12-07

**Authors:** Mara Atherton, Aleeza Safdar, Rouzbeh Motiei-Langroudi

**Affiliations:** 1 Medicine, University of Kentucky, Lexington, USA; 2 Neurosurgery, University of Kentucky, Lexington, USA

**Keywords:** adjacent segment pathology, pseudoarthiritis, short-segment posterior lumbar fusion, interbody device, posterior lumbar fusion, outcome, interbody, fusion, lumbar, posterior

## Abstract

Introduction: Interbody devices (IBDs) have been shown to improve outcomes when used in posterior lumbar fusion (PLF) surgery; however,the exact extent of their clinical benefit remains a current topic of interest. Our primary objective in this study was to identify whether the use of an IBD at every level of fusion construct would affect fusion outcomes such as adjacent segment pathology (ASP) and pseudarthrosis after one- to three-level PLF surgery.

Methods: This was a single-institution retrospective study. We studied the association of factors such as smoking status, BMI, gender, age, and number of IBDs on the development of ASP and pseudarthrosis. To study the effect of independent variables on ASP and pseudoarthrosis, univariate and multivariate regression analyses were used.

Results: The study included 2,061 patients with a history of posterior lumbar fusion who were identified and reviewed. Among these, 363 patients met our inclusion criteria; 247 patients had a minimum follow-up of six months and were finally included in the study. The median follow-up was 30 months. Among the 247 patients, 105 (42.5%) and 24 (9.7%) experienced ASP and pseudarthrosis, respectively. Gender and use of IBD significantly affected the presence of pseudarthrosis (with a higher rate in males and those without any IBDs). Gender, age, BMI, and use of IBDs did not affect ASP. Moreover, using an IBD at each fused level reduced the pseudarthrosis rate significantly compared to when IBDs were not used at all levels (7.3% vs. 27.6%, p <0.001), while there was no significant difference in the rate of ASP (43.6% vs. 34.5%, p = 0.35).

Conclusions: In patients undergoing one- to three-level PLF surgery, the use of an IBD at all levels of the fusion construct significantly reduces the rate of pseudarthrosis. There was no significant correlation between the rates of ASP. Studies with a larger sample size and a longer follow-up time are suggested to validate our results for pseudoarthrosis and ASP. Our results suggest the use of an IBD per fusion level in short-segment PLF surgeries.

## Introduction

Posterior lumbar fusion (PLF) is a widely used technique for the treatment of various diseases of the lumbar spine, such as spinal stenosis, scoliosis, or spondylolisthesis. This can be accomplished through instrumentation involving either pedicle screws, interbody devices (IBDs), a combination of the two, or other techniques. Although IBDs have been shown to add benefits when included in PLF, such as decreased pseudarthrosis rate, improved sagittal balance, and decreased reoperation rates [1-6], the exact extent of their clinical benefit remains a current topic of interest, as most of the published articles suffer from small sample sizes, inadequate length of follow-up, or use a mix of fusion techniques studied instead of focusing on a single surgical approach [6-10]. Furthermore, whether the number of IBDs should be matched to all levels of screw fixation (i.e., use an IBD for every level fused with pedicle screws) remains unknown [9-11].

In this study, we investigated whether the number of levels of IBDs affects fusion outcome, particularly the occurrence of pseudarthrosis and adjacent segment pathology (ASP) in patients who underwent short-segment (one- to three-level) PLF surgery. We particularly analyzed whether the rates of ASP, pseudarthrosis, and re-operation were different or not in patients having an IBD at each level fused with pedicle screws compared to those who had fewer IBDs.

## Materials and methods

This is a retrospective study of patients who underwent one-, two-, or three-level PLF surgery at a single academic institute, The University of Kentucky Chandler Hospital, Lexington, Kentucky, United States, from 2010 to 2020. All adult patients who had degenerative spine pathologies such as stenosis, spondylolisthesis, and disc herniations and had undergone PLF surgery at levels L2-L3, L3-L4, and L4-L5 were retrospectively reviewed. All patients included were refractory to conservative management such as physical therapy and steroid injections. Patients who had non-degenerative pathologies (such as spine tumors, infections, or trauma), those fused with stand-alone IBDs, or those who had a history of any previous lumbar spine surgery (such as a micro-discectomy, laminectomy, fusion, etc.) were excluded. Patients who had surgery at levels L5-S1, L1-L2, or above or had a surgical approach other than posterior (anterior, oblique, or lateral) were also excluded. Patients with surgery at the L1-L2 level and L5-S1 level were excluded due to a known high rate of pseudarthrosis reported at these levels.

The following demographic and surgical variables were recorded by reviewing the medical and billing records: age, gender, BMI, smoking status, indication for surgery, surgical details (including number of levels fused with pedicle screws, levels fused with IBDs, and the surgical approach), follow-up length, clinical outcome at last follow-up, fusion outcome, date of re-operation, indication for re-operation, and clinical outcome after re-operation. The presence of pseudarthrosis and ASP was determined by the clinic and operative notes of the treating surgeon and confirmed by imaging findings (MRI and CT scans). Cage subsidence in the absence of bony fusion was considered pseudarthrosis. Adjacent segment pathology was diagnosed by the presence of clinical symptoms such as neurogenic claudication and radicular or low back pain and confirmed radiographically by the presence of new imaging findings such as new stenosis, spondylolisthesis, disc degeneration, and facet changes at one or two levels above or below the operated levels on MRI. Pseudarthrosis was diagnosed by the presence of radicular or low back pain and confirmed radiographically on CT scan by the presence of radiolucency around fusion material (pedicle screws or interbody cages) and a lack of formation of bridging posterolateral bone, as confirmed by positive intra-operative findings. Throughout this manuscript, whenever all the levels were fused with both pedicle screws and IBDs, it will be described as “matching” (i.e., whenever surgery was at L3-L4 and L4-L5, patients received pedicle screws in L3, L4, and L5 and IBDs at L3-L4 and L4-L5, and so on). When fewer levels were fused with IBDs than pedicle screws (i.e., when surgery was at L3-L4 and L4-L5 and patients received pedicle screws in L3, L4, and L5, but they received no IBDs or at most one, either at L3-L4 or L4-L5), it will be described as "non-matching." The interbody cages used were similar across the cohort for all patients.

The data were analyzed using IBM SPSS Statistics for Windows, Version 28.0 (IBM Corp., Armonk, NY). Qualitative and quantitative data were analyzed using chi-square and independent t-test tests, respectively. To predict the effect of independent variables on outcomes, multivariate regression analysis was used with variables that had a p-value less than 0.1 on univariate analysis (chi-square and t-tests). P-values <0.05 were considered statistically significant. The University of Kentucky's Institutional Review Board approved this study (approval number: 63666).

## Results

A total of 2,061 medical records were initially identified and reviewed. Out of these, 363 patients met our inclusion criteria. When limiting the minimum follow-up to six months, 247 patients remained in the study. Among these, 206, 145, and 12 patients had one-, two-, and three-level PLF surgery, respectively. There was a minimum follow-up of two years for 138 patients and a minimum follow-up of three years for 100 patients.

For all patients, static (non-expandable) polyether-ether-ketone (PEEK) IBDs were used. Bone morphogenetic protein (BMP) was not used in any patient. At the time of the index operation, the mean age (SD) was 62.7+10.0 years, and the BMI was 30.7+6.1 kgm^2^. The female-to-male ratio and rate of tobacco use (current smoker at the time of surgery) were two and 16.2%, respectively. The follow-up length range was from six to 129 months. Among the 247 patients, 105 (42.5%) and 24 (9.7%) experienced ASP and pseudarthrosis, respectively. The location of ASP occurrence relative to the fusion construct is displayed in Table 1.

**Table 1:** Frequency of adjacent segment pathology (ASP) occurrence at vertebral levels relative to the construct

Location of ASP occurrence relative to construct	N (%)
2 levels rostral	1 (1.0%)
1 level rostral	68 (64.8%)
1 level caudal	15 (14.3%)
Multiple levels	21 (20.0%)
Total	105 (100%)

Univariate analysis showed that gender and use of IBD affected the presence of pseudarthrosis (with a higher rate in males and those without any IBDs), while age and BMI did not. Accordingly, gender, age, BMI, and use of IBDs did not affect ASP, as displayed in Table 2.

**Table 2:** Factors predicting the occurrence of pseudarthrosis and ASP BMI: body mass index; ASP: adjacent segment pathology; IBD: interbody device; matching IBD: use of an IBD for every level fused with pedicle screws Each cell represents mean±SD for quantitative variables and percent for qualitative variables.

	Pseudarthrosis			ASP			
	Yes (n=24)	No (n=223)	p-value	Yes (n=105)	No (n=142)		p-value
BMI, (kg/m^2^)	28.6 ± 5.6	30.9 ± 6.1	0.08	30.0 ± 5.2	31.2 ± 6.7		0.11
Age, (years)	61.4 ± 8.2	62.8 ± 10.2	0.52	62.7 ± 9.3	62.6 ± 10.6		0.92
Gender			0.02*				0.48
Female	6.6%	93.4%		41.0%	59.0%		
Male	16.0%	84.0%		45.7%	54.3%		
Tobacco			0.22				0.48
Smoker	15.0%	85.0%		37.5%	62.5%		
Non-smoker	8.7%	91.3%		43.5%	56.5%		
Use of IBD			0.02*				0.25
Yes	8.5%	91.5%		43.4%		56.6%	
No	33.3%	66.7%		25%	75%		
Matching IBDs			<0.001*				0.43
Yes	7.3%	92.7%		43.6%	56.4%		
No	27.6%	72.4%		34.5%	65.5%		

It was shown in the multivariate regression analysis that there was a significant correlation between the independent variables and pseudarthrosis (p <0.001; Hosmer-Lemeshow goodness of fit: R-square = 17.35, p = 0.03). Non-matching IBDs predicted pseudarthrosis (odds ratio (OR) = 3.8; p = 0.02), while factors such as age, sex, BMI, and tobacco use did not significantly predict pseudoarthrosis (p = 0.58, 0.09, 0.10, and 0.38, respectively). Regarding ASP, the variables in the model also significantly predicted outcome (p = 0.02; Hosmer-Lemeshow goodness of fit: R-square = 17.56, p = 0.03). The only statistically significant predictor was follow-up length (p <0.001), while age, gender, BMI, tobacco use, total number of levels fused, and presence of matching IBDs were not significant predictors of ASP (p = 0.81, 0.10, 0.09, 0.92, 0.44, and 0.45, respectively).

Effect of the number of IBDs on the fusion outcome

Analysis showed that the number of IBDs affected the surgical outcome. The presence of matching IBDs reduced the pseudarthrosis rate significantly compared to non-matching fusions (7.3% vs. 27.6%, OR = 12.0, p <0.001), while there was no significant difference in the rate of ASP (43.6% vs. 34.5%, p = 0.35), as shown in Table 2. We further broke down the effect of the number of IBDs on outcomes for single-level and two-level PLFs. Results showed that the presence of matching IBDs reduced the pseudarthrosis rate significantly while having no significant effect on ASP, both in one- and two-level PLF (Table 3).

**Table 3:** Rate of pseudarthrosis and adjacent segment pathology (ASP) for one- and two-level fusions ASP: adjacent segment pathology; IBD: interbody device; matching IBD: use of an IBD for every level fused with pedicle screws Data for three-level fusion were not presented due to a low number of patients.

Level of fusion						
Patients with one-level fusion	Pseudarthrosis			ASP		
	Yes	No	p-value	Yes	No	p-value
Matching IBDs			0.09			0.95
Yes	5.7%	94.3%		35.6%	64.4%	
No	18.2%	81.8%		36.4%	63.6%	
Patients with two-level fusion	Pseudarthrosis			ASP		
	Yes	No	p-value	Yes	No	p-value
Matching IBDs			0.001*			0.22
Yes	4.6%	95.4%		23.1%	76.9%	
No	40%	60%		0%	100%	

For single-level fusions, multivariate regression analysis showed that there was a significant correlation between the independent variables and pseudarthrosis (p <0.001; Hosmer-Lemeshow goodness of fit: R-square = 12.00, p = 0.15). Non-matching IBDs and lower BMI predicted pseudarthrosis, while factors such as age, sex, and tobacco use did not significantly predict pseudarthrosis (all p-values above 0.05). Regarding ASP, the variables in the model also significantly predicted outcome (p <0.001; Hosmer-Lemeshow goodness of fit: R-square = 9.04, p = 0.34). The only statistically significant predictor was BMI (p <0.03), while age, gender, tobacco use, total number of levels fused, and presence of matching IBDs were not significant predictors of ASP (all p-values above 0.05). For two-level fusions, multivariate regression analysis showed that there was a significant correlation between the independent variables and pseudarthrosis (p <0.001; Hosmer-Lemeshow goodness of fit: R-square = 6.75, p = 0.56). Only non-matching IBDs predicted pseudarthrosis, while factors such as age, sex, BMI, and tobacco use did not significantly predict ASP (all p-values above 0.05). Regarding ASP, the variables in the model also significantly predicted outcome (p <0.001; Hosmer-Lemeshow goodness of fit: R-square = 8.30, p = 0.41). Only non-matching IBDs predicted ASP, while factors such as age, sex, BMI, and tobacco use did not significantly predict ASP (all p-values above 0.05).

Effect of the number of IBDs on the fusion outcomes in patients with follow-up lengths greater than two and three years

Among the 138 patients with a follow-up length of at least two years (24 months), 19 (13.8%) and 88 (63.8%) developed pseudarthrosis and ASP, respectively. Since ASP takes at least three years to develop, we separately looked at the 100 patients with a follow-up length of at least three years (36 months), and out of those, 10 (10.0%) and 71 (71.0%) developed pseudarthrosis and ASP, respectively. The influence of the use of IBDs (matching vs. non-matching) in this subset of patients is shown in Table 4.

**Table 4:** Effect of the number of interbody devices on fusion outcomes in patients with a minimum follow-up of two to three years ASP: adjacent segment pathology; IBD: interbody device; matching IBD: use of an IBD for every level fused with pedicle screws

Follow-up length						
Follow-up longer than two years	Pseudarthrosis			ASP		
	Yes	No	p-value	Yes	No	p-value
Matching IBDs			0.002*			0.28
Yes	10.1%	89.9%		65.5%	34.5%	
No	36.8%	63.2%		52.6%	47.4%	
Follow-up longer than three years	Pseudarthrosis			ASP		
	Yes	No	p-value	Yes	No	p-value
Matching IBDs			0.002*			0.41
Yes	6.0%	94.0%		72.6%	27.4%	
No	31.3%	68.7%		62.5%	37.5%	

Results showed that a matching number of IBDs significantly decreased pseudarthrosis in both follow-up subsets while not influencing ASP, even when the minimum follow-up was reduced to three years.

## Discussion

Posterior lumbar fusion is a widely used technique for lumbar fusion. Frequently, PLF is combined with the insertion of IBDs for surgical benefits such as improved lordotic angle via anterior column support and greater surface area for the fusion interface. Previous studies have shown that adding IBDs to PLF increases the rate of fusion compared to fusion with screws alone [3, 8, 10-12]. In a meta-analysis, Levin et al. showed that PLF with screws alone was found to have lower odds of achieving a solid arthrodesis (OR = 0.33; 95% CI 0.13-0.82; p = 0.02) compared to the use of screws plus IBDs [11].

Our study confirmed these results by showing that the use of IBDs in general decreased the rate of pseudarthrosis while having no negative influence on ASP. Moreover, we showed that when IBDs were included at every level of the fusion construct, the rate of pseudarthrosis was significantly less compared to the constructs that did not have IBDs at every level. To our knowledge, this is the first time that this finding has been shown in the literature. One biomechanical explanation for this finding is that adding an adequate number of IBDs shares the load with screws, increases support of the anterior column postoperatively, and results in a higher fusion rate. However, the suggestion to use one IBD per fusion level in one- to three-level PLFs should be carefully considered by practicing surgeons, as more IBDs directly translate into longer operative times, more blood loss, added cost, and theoretically higher complication rates (nerve root injury, incidental durotomy, etc.). Our results showed that the male gender was associated with higher rates of pseudarthrosis than the female gender, which is contrary to what has been reported previously. A meta-analysis published in 2019 by Liu et al. found that gender was unlikely to be associated with any differences in pseudarthrosis rate from cage retropulsion [13]. One explanation for our finding can be that the occupational differences between males and females in the study population that may have confounded our results, as again, the difference was not confirmed by multivariate analysis.

Our study is limited by its retrospective nature. Different surgeons were involved over the course of 10 years, and surgeon-related factors have not been reported as they are not the primary focus of our study, and they are difficult to acknowledge in a retrospective review. While nowadays widely used in our center, patient-reported outcome measures (PROMs) were unavailable for this patient population and have not been reported. We are also limited by a small sample size when it comes to sub-dividing the patients into one-, two-, or three-level PLF surgery and comparing outcomes. Knowing the benefits of prospective studies, performing one with adequate follow-up length is difficult in spine surgery. Our average follow-up was 38 months (slightly more than three years). Adjacent segment pathology takes longer to present than pseudarthrosis (average of three years vs. three to six months). A follow-up of at least three to 10 years is needed to fully investigate the effect of IBD on ASP. However, we believe our conclusions are more accurately reflective of the effect of IBDs on reducing rates of pseudoarthrosis after PLF.

## Conclusions

In patients undergoing one- to three-level PLF surgery, the use of an IBD at all levels of the fusion construct significantly reduces the rate of pseudarthrosis. There was no significant correlation between the rates of ASP. Studies with a larger sample size and a longer follow-up time are suggested to validate our results for pseudoarthrosis and ASP. Our results suggest the use of an IBD per fusion level in short-segment PLF surgeries.
